# Co-endemicity of Loiasis and Onchocerciasis in Rain Forest Communities in Southwestern Nigeria

**DOI:** 10.1371/journal.pntd.0003633

**Published:** 2015-03-26

**Authors:** Olusola Ojurongbe, Akeem Abiodun Akindele, Monsuru Adebayo Adeleke, Matthew Oyebode Oyedeji, Samuel Adeyinka Adedokun, Josephine Folashade Ojo, Callistus Adewale Akinleye, Oloyede Samuel Bolaji, Olusegun Adelowo Adefioye, Oluwaseyi Adegboyega Adeyeba

**Affiliations:** 1 Department of Medical Microbiology & Parasitology, Ladoke Akintola University of Technology, Osogbo, Nigeria; 2 Department of Community Medicine, Ladoke Akintola University of Technology, Osogbo, Nigeria; 3 Department of Biological Sciences, Osun State University, Osogbo, Nigeria; University of Cambridge, UNITED KINGDOM

## Abstract

**Background:**

Loiasis is currently receiving attention as a disease of public health importance because of the possibility of increased risk of developing neurologic serious adverse event following mass ivermectin treatment against onchocerciasis in individual co-infected with *Onchocerca volvulus* and *Loa loa*.

**Methodology/Principal Findings:**

Rapid assessment procedure for loiasis (RAPLOA) was conducted in 12 communities covering the 3 senatorial districts of Osun State, Nigeria. A total of 960 people were interviewed for history of eye worm using the WHO guidelines for rapid assessment. The survey confirmed the presence of loiasis in all the 12 communities with 4 in Osun East/Ife south senatorial district being at high risk with a prevalence of over 40%. Based on the RAPLOA results, communities within Osun East/Ife south senatorial district were selected for microfilaraemic assessment of *L*. *loa* and *O*. *volvulus*. A total of 1115 and 1091 individuals were screened for *L*. *loa* and *O*. *volvulus* microfilaria worms respectively. 160 (14.3%) had *L*. *loa* microfilaria detected in their blood with 8 (5.0%) individuals having *L*. *loa* loads above 8000 mf/ml. 166 (15.2%) subjects had *O*. *volvulus* microfilaria (range 4-504 mf/ml) detected in their skin snip. 30 (2.69%) subjects were co-infected with both *L*. *loa* and *O*. *volvulus*. There was a significant variation in the prevalence (2.1% to 33.3%) of onchocerciasis in the communities studied (p = 0.001). Five (41.7%) of the studied communities had a prevalence that is equal to or greater than 20%.

**Conclusions/Significance:**

Low prevalence of onchocerciasis and loiasis co-infection in this study suggests that loiasis may not pose a serious epidemiological threat to the continuous distribution and sustainability of ivermectin for the treatment of onchocerciasis. Evaluation of the interruption of onchocerciasis transmissions in this region using all the indicators set forth by WHO is therefore suggested.

## Author Summary

Loiasis recently emerged as a disease of public health importance because of the risk of rare severe adverse events (SAEs) associated with the rapid killing of *Loa loa* microfilariae in heavily parasitized individuals following ivermectin treatment. for onchocerciasis. The possibility of developing these SAEs has been slowing down the control of onchocerciasis in some endemic communities. Based on information available at the Osun State Ministry of Health, the detailed geographic distribution of *L*. *loa* in Osun State is still awaiting elucidation. The results of this study revealed an overlap in geographic distribution of loasis and onchocerciasis in many communities in Osun State. The overall Community Microfilaria Load (CMFL) for *L*. *loa* was below the threshold level of endemicity that could be associated with the risk of SAEs after ivermectin treatment. Most individuals with slightly high *L*. *loa* microfilaria are either *O*. *volvulus* microfilaria free or have very low microfilaria load. The low prevalence of co-infection of both diseases in the study area is an indication that loiasis may not pose any serious epidemiological threat to the distribution and sustainability of ivermectin in the treatment of onchocerciaisis.

## Introduction

Onchocerciasis and loiasis are endemic filarial parasites in the central and western African countries [1] including Nigeria. Onchocerciasis is rated as the second leading infectious cause of blindness in the world only preceded by blinding trachoma [2,3]. It is endemic in 31 countries in Africa, the Arabian Peninsula and 6 in the Americas. 30 out of 36 endemic countries are in sub-Sahara African countries where approximately 99% of all those infected live [2]. Nigeria probably has the highest burden of onchocerciasis in the world, accounting for about a third of the global prevalence [4,5]. The disease has had a major impact on the economic and social lives of the endemic communities [6]. Loiasis, has recently emerged as a disease of public health importance, not because of its own clinical manifestations only but also because of the concern about neurological complications that may occur when ivermectin is given as part of mass treatment programmes for onchocerciasis to people co-infected with *Onchocerca volvulus* and *Loa loa* [7]. It is an African disease restricted to the equatorial rain forest regions of Central and West Africa. *L*. *loa* is often referred to as the African eye worm because the adult worm can sometimes be seen moving through the sclera of the eye [8].

The control of onchocerciasis in endemic African countries is coordinated by African Program on Onchocerciasis Control (APOC) through annual distribution of ivermectin the drug of choice for the control of onchocerciasis, donated free by Merck & Co. and is being distributed through community directed approach. This approach has recorded a huge success which has led to the suggestion that onchocerciasis is currently in the elimination phase in most countries that commenced ivermectin distribution over 15 years ago [9]. *Loa loa* causes a chronic infection in humans that is characterized by two clinical features which are tropical "eye worm" (migration of adult worms across the sub-conjunctiva) and Calabar swelling, (localized angioedema found predominantly on the extremities). It is transmitted by species of horse-flies (*Chrysops* spp.), most commonly *Chrysops dimidiate* and *C*. *silacea* which inhabit the forest areas of West and Central Africa, extending to the Ethiopian border [10].

Cases of Serious Adverse Experiences (SAEs) have been observed in individuals with very high *L*. *loa* microfilarial loads (more than 30,000 microfilariae (mfs) per ml of blood) who had been treated with ivermectin in onchocerciasis endemic communities [11]. The large-scale distribution of ivermectin was successfully introduced into endemic communities until neurologic serious adverse events (SAEs) in individuals with high *L*. *loa* microfilaraemia after ivermectin treatment were reported [12][13][14]. *Loa loa* infection is a serious constraint to mass treatment for onchocerciasis in parts of 11 African countries, where APOC estimates about 14.3 million persons to be at risk of such serious adverse events [15]. This has necessitated the need to always identify those areas where loiasis is endemic, and where specific monitoring procedures should be developed along with large-scale ivermectin treatment of onchocerciasis.

According to the rapid epidemiological mapping of onchocerciasis in Nigeria in 1995 and 1996, [16], Osun State is mesoendemic for onchocerciasis and community directed mass ivermectin treatment has started in the State since year 2000. The drug is presently being distributed in all the sixteen endemic Local Government areas of the State. Reports from our personal interaction with some endemic communities revealed that there are few individuals who have not been taking ivermectin for some years now owing to severe reactions to the drug. To the best of our knowledge and based on information available at the State Ministry of Health (SMOH) in Osun State, the detailed geographic distribution of *L*. *loa* in Osun State is still awaiting elucidation as previous survey available at SMOH was skewed to few communities leaving larger proportion of the State uncovered. The paucity of information on *Loa loa* may undoubtedly, remain a major impediment for the realization of community coverage threshold and sustainability of ivermectin distribution in the endemic communities in the State. Therefore, there is need for mapping the rural communities in other to identify the co-existence of *L*. *loa* and *O*. *volvulus*. This will help in the development of safe treatment and sustainable distribution regime for the control and elimination of onchocerciasis in the State. In this study, we report the results of epidemiological studies on co-endemicity of onchocerciasis and loiasis in Osun State, South-western Nigeria.

## Methods

### Study site

**Fig 1:**
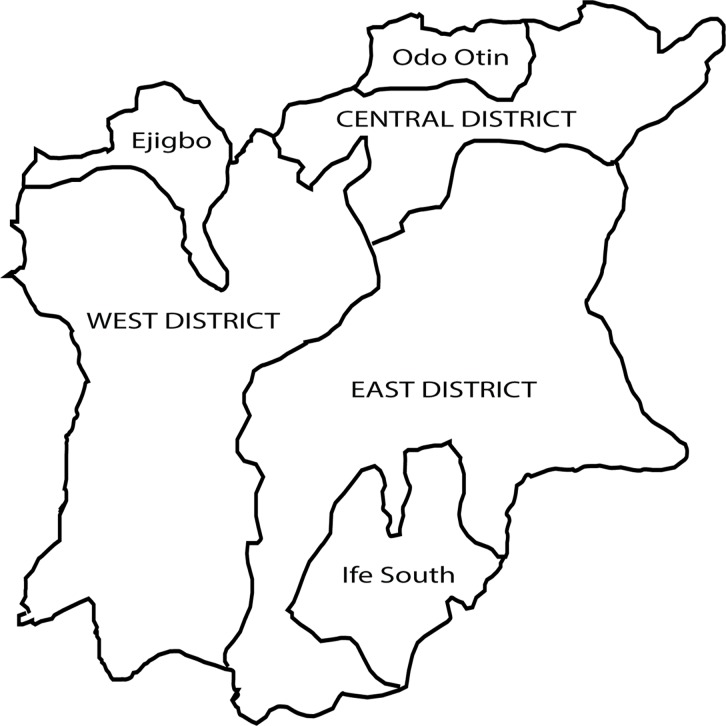
Map of Osun State showing the three senatorial districts and the communities within the districts that were selected for the study.

Osun State is situated in the tropical rain forest zone. It covers an area of approximately 14,875 sq km and lies between latitude 7° 30′ 0″ N and longitude 4° 30′ 0″ E. There are two seasons, the rainy season from April to October and the dry season from November to March. The state is covered by secondary forest and in the northern part, the derived Savannah mosaic predominates. Forest vegetation could be found along the river valleys and streams found across the State. The economy of Osun State is agro-based with farming being the predominant occupation of the people. Osun State comprises of 30 Local Government Areas (LGAs); geopolitically stratified into three Senatorial districts and each Senatorial district consists of at least 8 (LGAs) (Fig. 1). Rapid Epidemiological Mapping of Onchocerciasis (REMO) was conducted in the state in 1996 and annual mass ivermectin distribution started in 2000 and it is still ongoing. The treatment coverage since inception increased gradually from 42% in 2000 to 100% in 2008 [17] and this has been sustained in the state for the last four years. Following the REMO result, the state was classified as hyperdermic. Mass Ivermectin distribution started in 1998 and all the Local government areas of the State have been receiving annual mass ivermectin treatment till date. From a database of all endemic communities, a list was generated, out of which four communities in each senatorial district were randomly selected for RAPLOA by balloting (Table 1). Based on the outcome of the RAPLOA, Osun East senatorial district was found to be endemic and later selected for parasitological examination of *Loa loa* and *O*. *volvulus* infections. For the parasitological survey, 12 communities were then selected from Osun East senatorial district.

**Table 1:** Number of villages surveyed and number of people interviewed in 12 villages of Osun State for RAPLOA.

Senatorial District/LG	Village	Coordinates	No. Interviewed	No. with history of eye worm (%)
Osun East/Ife south	Bolounduro	7.025E; 4.630N	80	37 (46.3)
	Omidi Onipetesi	7.37549E; 4.547286N	80	54 (67.5)
	Nathaniel-Olodo	7.39916E; 4.56961N	80	41 (51.3)
	Odemuyiwa	7.30177E; 4.60623N	80	40 (50.0)
Osun West/Ejigbo	Owu Ile	7.7842543E; 4.1472789N	80	13 (16.3)
	Ife-Odan	7.82549E; 4.13395N	80	16 (20.0)
	Inisha	7.84974E; 4.33287N	80	35(43.8)
	Isoko	7.91674E; 4.28205N	80	29(36.3)
Osun Central/Odo Otin	Oore	7.96706E; 4.56633N	80	20 (25.0)
	Opete	8.06746E; 4.61667N	80	26 (32.5)
	Ila-Odo	8.06746E; 4.69872N	80	19 (23.8)
	Imuleke-Oponda	8.0453531E; 4.690607N	80	14(17.5)

### Ethical consideration

Ethical clearance was obtained from the Osun State University College of Health Science ethical review committee. The Ethical review committee approved oral consent based on the premise that participants are not literate. Permission was also obtained from the paramount rulers and the respective heads of the communities where the investigations were undertaken. Oral informed consent was obtained from all consenting individuals that participated in the study and from parents/guardians of those less than 18 years. Each adult above the age of 18 years and parents/guardians of children were individually briefed on the objectives of the survey and informed that they were free to participate or refuse. Consent was recorded as ‘yes’ or ‘no’ on an individual form designed for the collection of basic demographic data. For those who refused to participate no further questions were asked and no information was recorded.

### Survey procedures for RAPLOA

The surveys were conducted using the RAPLOA methodology as described in the guidelines for rapid assessment of *L*. *loa* [15]. This methodology consists of three steps:

1. identification of local names for the *L*. *loa* eye worm using a community-level questionnaire;
2. collection of information on the history of eye worm, from adults in the community, using an individual-level questionnaire which has three key questions;
3. calculation of the percentage of adults who report a history of eye worm, and, on the basis of this percentage, prediction of the level of *L*. *loa* endemicity.

At the beginning of the RAPLOA survey in each community, the questionnaire was administered to key informants (village heads, schoolteachers, health workers, patent medicine dealers, traditional healers, and women and group leaders) to determine the local names for the eye worm, the population size and the number of households in the community. After administration of the community questionnaire, the geographic coordinates (latitude and longitude) of the community were collected using a geographical positioning system (GPS) unit in a central point or in front of the house of the community chief. All adults in the community fulfilling the criteria for inclusion (aged 15 years and above and/or residents in the community for at least 5 years) were included until the required number of 80 individuals per community has been reached.

The individual questionnaire was designed to elicit responses on experience of eye worm. Three key questions were asked chronologically to collect data on the experience of eye worm. The first question in each interview was ‘‘Have you ever experienced or noticed worms moving along the white of the lower part of your eye?”. After recording the response, the interviewer then showed a photograph of the eye worm to each respondent, guided him/her to recognize the worm on the photograph and then asked the second question: ‘‘Have you ever had the condition in this picture?”. After recording the answer, the interviewer proceeded to ask the third question: ‘‘the last time you had this condition, how many days did the worm last before disappearing?”. Other forms of eye discomfort like red eye, pain in the eye and blurry visions were also noted.

A respondent was classified as having a history of eye worm when the answers to the first two questions were positive and the duration in the third question was less or equal to 7 days. For each community the percentage of respondents with a history of eye worm was computed to give the prevalence of history of eye worm.

### Sample collection

The respective community heads assisted to mobilize their subjects in the respective communities. Information on each individual was obtained using well-structured questionnaires. Information like age, sex, occupation, clinical symptoms, history of treatment with mectizan, and duration of stay within the community was obtained. Venous blood samples and skin snips were collected from consenting residents aged 5 to 80 years, and sera were obtained from the clotted blood. All parasitological assessments were conducted six months post ivermectin distribution in the selected community.

### Skin snip examination for *Onchocerca volvulus*

Two skin snip was taken from the two iliac crests of each study participant under aseptic condition using disposable sterile pricking needle and razor blade. The skin snips were taken from each participant and then separately placed in flat-bottomed micro-titre plate wells filled with 100 μl of physiological saline and left at room temperature for 24 hours. The identity of each participant was labelled on each microtitration plate. The whole contents in individual well was transferred onto a microscopic slide. The emerged microfilariae were counted under 10x microscope objective. The Community Microfilarial Load (CMFL) was calculated as the geometric mean of microfilarial load per community [18].

### Blood examination for *Loa loa* microfilariae

The venous blood was collected from each participant during the day time (between 10:00am and 16:00pm) and microfilariae were examined directly in a wet blood film between microscope slide and coverslip, using an optical microscope. The blood films were fixed in methanol, stained with haematoxylin and examined microscopically for microfilariae. Microfilariae loads were assessed quantitatively by calibrated blood smears [19]. Parasitemia were expressed in microfilariae per milliliter (mf/ml) of blood.

### Data analysis

Data were entered into a Microsoft Excel database (Microsoft Corporation, Seattle, WA) subject to validation checks. The data were then analyzed using SPSS 17 for windows (2007 version). The microfilarial load of each subject was estimated as the mean number of microfilariae per skin snipped. The mean microfilarial load was calculated for all positive participants. The CMFL was obtained as the geometric mean of microfilarial load for participants. Student’s t-test was used to determine significant differences in the intensity of infection across age groups. Differences in proportions were tested using chi-square (χ^2^) test. P values < 0.05 were considered statistically significant.

## Results

A total number of 960 people were interviewed for their history of eye worm. Table 1 gives the number of communities surveyed, the number of individuals examined and the percentage of communities that exceeded the thresholds of 40% for RAPLOA. The surveys confirmed the presence of loiasis in all the twelve communities, however, only four communities were high risk communities where over 40% of those interviewed reported a history of eye worm. Osun East senatorial district recorded the highest number of people with history of eye worm.

Following the RAPLOA survey Osun East senatorial district was subsequently selected for microfilaraemic assessment. A total of 1,115 individuals (mean age 46.0 years (range: 5–110 years); 665 (59.6%) females; 450 (40.4%) males) were microscopically examined for *L*. *loa* and 1093 examined for *O*. *volvulus* infections in Osun East senatorial district. Table 2 shows the general characteristics and distribution of filarial infections among the study population. In all, 160 (14.3%) had *Loa loa* detected in their blood while 166 (15.2%) subjects had *O*. *volvulus* microfilaria detected in their skin snip. A total of 293 (26.3%) harboured at least either *Loa loa* or *O*. *volvulus* while 30 (2.69%) were co-infected with both *L*. *loa* and *O*. *volvulus*.

**Table 2:** General characteristics and distribution of filarial infections among the study population.

Patient’s characteristics	No. for ***Loa loa*** (%)	No. for Onchocerciasis (%)
Number of participants examined	1115	1091
Male: Female	450: 665	450: 641
Mean age (years)	46.00	45.74
No. Positive for microfilaria (%)	160 (14.30)	166 (15.20)
Co-infection of *O*. *volvulus* + *Loa loa* (%)	30(2.69)	30(2.75)
Cases of eye worm among the population	485 (43.50)	401 (36.80)
Nodules carriers among the population	64 (5.74)	64 (5.87)

Table 3 shows community by community prevalence of *L*. *loa* and *O*. *volvulus* infection. The results revealed a significant variation in the prevalence of onchocerciasis in the communities studied (p = 0.001). The prevalence of *O*. *volvulus* infection ranged from 2.1 to 33.3% with an overall prevalence of 15.2%. Five (41.7%) of the studied communities had a prevalence that is equal to or greater than 20%. Alaba metta had the highest prevalence (33.3%) while the lowest was found at Aye Balogun (2.1%). The CMFL of *O*. *volvulus* similarly showed significant inter- community variations at the study area. All the communities had less than one microfilariae per skin snip.

**Table 3:** The prevalence of loiasis and onchocerciasis in the selected rural communities in Osun State.

Villages	Loiasis					Onchocerciasis		
	No, Examined	No. Positive	No. <8000mf/ml (%)	No. >8000mf/ml (%)	CMFL	No. Examined	No. Positive	CMFL
Ajebamidele	233	18 (7.7)	18 (100)	-	1.342	209	50 (23.9)	0.157
Alaba metta	84	3 (3.6)	3 (100)	-	1.637	84	28 (33.3)	0.398
Amula Saliu	54	15 (27.8)	15 (100)	-	1.459	54	9 (16.7)	0.125
Araromi Awosiyan	44	2 (4.5)	2 (100)	-	1.494	44	9 (20.5)	0.142
Atere	63	4 (6.3)	4 (100)	-	1.529	63	2(3.2)	0.048
Aye Balogun	145	28 (19.3)	25(89.3)	3(10.7)	2.243	145	3 (2.1)	0.030
Bolorunduro	75	7 (9.3)	7 (100)	-	1.809	75	15 (20.0)	0.085
Idi Ogun Adedire	60	11 (18.3)	11 (100)	-	1.494	60	10 (16.7)	0.141
Mefoworade	101	10 (9.9)	8 (80.0)	2(20.0)	1.459	101	22(21.8)	0.211
Nathaniel/ Olodo	62	18 (29.0)	18(100)	-	1576.	62	2(3.2)	0.113
Omidi Onipetesi	69	29 (42.0)	28(96.6)	1(3.4)	1.494	69	5 (7.2)	0.142
Amado	125	15 (12.0)	13(86.7)	2(13.3)	1.735	125	11(8.8)	0.159
Total	1115	160 (14.4)	152(95.0)	8(5.0)		1091	166 (15.2)	

Distribution of *Loa loa* microfilaraemia and *O*. *volvulus* by gender and age is shown in Table 4. While no significant difference was observed in both *L*. *loa* and *O*. *volvulus* infection with respect to gender, a significant difference was observed in *L*. *loa* infection with respect to age with the older age group (65 years and above) having the highest prevalence (p = 0.001). Also out of the 43 participants within the age group of 5–10 years, 5 (11.6%) were positive for *O*. *volvulus* microfilariae.

**Table 4:** Distribution of *Loa loa* microfilaraemia and *O*.*volvulus* by age and sex.

		No examined for *Loa loa*	No +ve for *Loa loa* (%)	No examined for oncho	No +ve for oncho (%)
SEX	Male	450	74 (16.4)	450	77 (17.1)
	Female	665	86 (12.9)	641	89 (13.9)
P-value			0.101		0.144
Age (yrs)	5–24	164	12 (7.3)	164	33 (20.1)
	25–44	351	40 (11.4)	345	51 (14.8)
	45–64	359	62 (17.3)	349	45 (12.9)
	65–84	204	44 (21.6)	199	31 (15.9)
	>84	37	2 (5.4)	34	6 (17.6)
Total		1115	160	1091	166
P value			0.001		0.368

The clinical manifestation observed in the study populations and its distribution between onchocerciasis and Loa loa positive members of the communities are shown in Table 5. History of eye worm (36%) and Calabar swelling (27.8%) were the most frequently observed clinical manifestations in the study population with 65.7% and 56% respectively of those with *Loa loa* microfilaria presenting with the symptom. Pruritus (22.2) was frequently more observed among onchocerciasis patients (90%) also eye lesion (7.5) was more common among onchocerciasis patients (20.6%).

**Table 5:** Clinical spectrum observed from filarial infected members of the surveyed population.

Clinical presentations	Total No. Observed n = 1115 (%)	No observed in Onchocerciasis (%) n = 160	No. observed in Loiasis (%) N = 166
Pruritus	248 (22.2)	144 (90.0)	58 (34.9)
Onchodermatitis	143 (12.8)	96 (60.0)	28 (16.9)
Eye discomfort	84 (7.5)	33 (20.6)	9 (5.4)
Nodules	64 (5.7)	23 (14.4)	18 (10.8)
History of eye worm	401 (36.0)	46 (28.8)	109 (65.7)
Calabar swelling	301(27.8)	30 (10.0)	93 (56.0)

## Discussion

For over 15 years, free mass treatment with ivermectin has been provided for the control of onchocerciasis and Osun State has been one of the beneficiary States in Nigeria. The community directed distribution of annual doses of ivermectin, coordinated by APOC is now moving from control to eradication phase in many endemic communities. One factor that has been slowing down the control of onchocerciasis in some endemic communities is serious adverse events (SAEs) in individuals with high *L*. *loa* microfilaraemia after ivermectin treatment [19][12]. Information on prevalence of loiasis and associated morbidities in onchocerciasis endemic areas therefore remains an important component needed for successful onchocerciasis elimination programme.

Based on available literature and information at the SMOH, this study is the first state-wide epidemiological report on loiasis and onchocerciasis co-infection in Osun State, Nigeria. The RAPLOA surveys represent a major effort of the APOC in response to a serious operational challenge for onchocerciasis control and lymphatic filariasis elimination. Out of the twelve communities selected from the three senatorial districts of the state for RAPLOA, 3(25%) communities, all located in Osun East Senatorial district had a prevalence of 40% and above (threshold value established by the RAPLOA development study). It is noteworthy that Osun/Ife East boarders Ondo State, Nigeria where high prevalence of *Loa loa* with intense biting of crysops in forest areas has been reported [20][21]. Also out of the twelve communities surveyed for *L*. *loa* microfilaria within Osun East Senatorial district, three of the communities had *L*. *loa* microfilareamia above 20% (threshold prevalence above which more than 5% of adults living in the community are at risk of adverse-related responses with ivermectin). To the best of our knowledge, no baseline survey was undertaken to determine the prevalence of *L*. *loa* before ivermectin distribution started in these communities some 12 years ago. From personal interview and community ivermectin records available, there has been no record of severe neurologic reactions capable of affecting the distribution of ivermectin in these communities; although there were some undocumented cases like dizziness, excessive scratching of the body and mild functional impairment which was described by personal interaction. One explanation for this observation is the fact that microfilaria load of *L*. *loa* observed in this study was very low. The highest intensity of *L*. *loa* infection in microfilaraemic patients observed in the study was 11500 mf/m. The overall CMFL for *L*. *loa* in the study was below the threshold level of endemicity that could be associated with the risk of neurologic serious adverse events after ivermectin treatment in a given area. Generally, it was observed that most individuals with slightly high *L*. *loa* microfilaria are either *O*. *volvulus* microfilaria free or with very low microfilaria load.

Although the total prevalence of 15.2% obtained shows that people still harbored *Onchocerca* microfilariae in the studied communities and there may be ongoing transmission of onchocerciasis in the communities; as five out of 43 participants within the age group of 5–10 years were positive for *O*. *volvulus* microfilariae. Though, the thrust of the current study was not geared towards the verification or establishment of interruption of onchocerciasis transmission in the communities, further studies considering all indicators set forth by WHO for certification of onchocerciasis interruption would be useful to measure the successes of ivermectin distribution in the studied communities. However, the results obtained on CMFL in this study presents us with an opportunity to evaluate the impact of the ivermectin on onchocerciasis in the studied communities. The CMFL obtained in the communities ranged from 0.030–0.398 microfilariae per skin snip which is below 5 microfilariae per skin snip set forth as a threshold value in considering onchocerciasis as a public health problem in a community. Onchocerciasis is only considered a public health problem when the CMFL exceeds 5 Mf/snip (WHO, 2001). Similar results had also been reported elsewhere in Southwestern Nigeria [18]. The low CMFL coupled with the current high treatment coverage (100%) (Njepuome et al., 2008) of ivermectin in most of the endemic communities in Osun State may be suggestive of feasibility of onchocerciasis elimination if treatment is sustained.

The low CMLF *O*. *volvulus* and *L*. *loa* obtained has many implications on the transmission and control of onchocerciasis in the area. The low density of microfilariae in human hosts usually reduces the vectorial potential of the *Simulium* vectors as the flies need to ingest relatively high number of microfilariae to ensure continuous transmission of infection [22]. Also the low CMFL of both *O*. *volvulus* and *L*. *loa* is an indication that the risk of serious adverse events after ivermectin treatment may be very low in these communities.

The prevalence of *L*. *loa* microfilaraemia was highest (14.3%) in the older people (65 - 84yrs). The high prevalence obtained in this age group may be the reflection of the exposure of this age group to *chrysop* bites than the younger groups since most of the older people are exclusively farmers. This may also explain the higher infection rate among the male subjects suggesting their higher level of exposure to the vectors especially through farming and other behavioral practices that could make them to be more prone to infection. In support of the result obtained, earlier studies in Nigeria had reported that males were likely to have higher prevalence of *L*. *loa* microfilaremia than females [23][24][21]. This assertion was also supported by the epidemiological data from central Cameroon where the prevalence of *L*. *loa* microfilaremia was significantly higher in male subjects than in females [19].

There is a good agreement between the results from the thick blood film microscopy and the results from RAPLOA. In line with the reports from other studies, clinical symptoms can be used to predict the impact and risk of side effects during mass chemotherapy [25][26]. As previously described, eye worm and Calabar swelling have been found to correlate strongly with prevalence of *L*. *loa* [15][27]. The overall prevalence of Calabar swelling in our study population was 27.8%. The swelling was observed in 56% of those positive for *L*. *Loa* microfilaria. This confirms the correlation and also confirms the endemicity of loaisis in these communities. Also high prevalence of pruritus was observed among individuals positive for onchocerciasis. Pruritus is a clinical sign of allergic reaction which is commonly observed in onchocerciasis endemic areas. Prevalence of itching has been shown to be significantly reduced following increased coverage of ivermectin. Subsequently we expect that these clinical symptoms will decrease significantly with effective coverage of ivermectin in a couple of years.

In conclusion, the results of this study revealed an overlap in geographic distribution of loasis and onchocerciasis in many communities in Osun State. There is a low prevalence of co-infection of both diseases in the study area which justifies the fact that loiasis may not pose serious epidemiological threat to the distribution and sustainability of ivermectin in the treatment of onchocerciaisis in the State. There is therefore need for pragmatic efforts aimed at achieving sustainable distribution of ivermectin towards eliminating onchocerciasis as a public health problem in the endemic communities, since CMFL obtained in the present study signifies the possibility of elimination in the study area.

## Supporting Information

S1 Checklist STROBE checklist. (DOC)
